# *Lactobacillus plantarum* WCFS1 and its host interaction: a dozen years after the genome

**DOI:** 10.1111/1751-7915.12368

**Published:** 2016-05-27

**Authors:** Maurits van den Nieuwboer, Saskia van Hemert, Eric Claassen, Willem M. de Vos

**Affiliations:** ^1^ Athena InstituteVrije UniversiteitAmsterdamThe Netherlands; ^2^ Winclove BV AmsterdamAmsterdamThe Netherlands; ^3^ Department of ViroscienceErasmus Medical CenterRotterdamThe Netherlands; ^4^ Laboratory of MicrobiologyWageningen UniversityWageningenThe Netherlands; ^5^ Department of Bacteriology & Immunology and Veterinary BiosciencesUniversity of HelsinkiHelsinkiFinland

## Abstract

*Lactobacillus plantarum* WCFS1 is one of the best studied Lactobacilli, notably as its genome was unravelled over 12 years ago. *L. plantarum* WCFS1 can be grown to high densities, is amenable to genetic transformation and highly robust with a relatively high survival rate during the gastrointestinal passage. In this review, we present and discuss the main insights provided by the functional genomics research on *L. plantarum* WCFS1 with specific attention for the molecular mechanisms related to its interaction with the human host and its potential to modify the immune system, and induce other health‐related benefits. Whereas most insight has been gained in mouse and other model studies, only five human studies have been reported with *L. plantarum* WCFS1. Hence NCIMB 8826 (the parental strain of *L. plantarum* WCFS1) in human trials as to capitalize on the wealth of knowledge that is summarized here.

## Introduction

There continues to be significant interest in lactic acid bacteria (LAB) that contribute to our quality of life by preserving and fortifying foods, producing flavours and texture, and providing health benefits (De Vos, 2011). Hence, recent years have seen the production of a panoply of publications that address these attributes in LAB with most of the applications relating to *Lactobacillus* spp. that are used in functional foods. While there are over 100 different *Lactobacillus* species that now all have been characterized at the genomic level (Sun *et al*., 2015), only few have been studied in detail and developed into paradigms, as is the case with many biotechnological systems. In 2003, the complete 3.3 Mb genome of *Lactobacillus plantarum* WCSF1, a single colony of the human saliva isolate *L. plantarum* NCIMB 8826, was published as the first genome of a *Lactobacillus* species (Kleerebezem *et al*., 2003). This was well before the genomes of other well‐studied *Lactobacillus* spp. were reported, such as those from *L. acidophilus* NCFM (Altermann *et al*., 2005) and *L. rhamnosus* GG (Morita *et al*., 2009) that are widely marketed as probiotics (Saxelin *et al*., 2005). Currently, there are 26 genome sequences of different *L. plantarum* strains available in the NCBI database. There is a high degree of gene content variation among *L. plantarum* strains (Molenaar *et al*., 2005). Due to the presence of its genome sequence, its flexible growth properties and high transformation efficiency with newly developed genetic tools (Pavan *et al*., 2000; Kleerebezem *et al*., 2003; Cohen *et al*., 2006; Yang *et al*., 2015), *L. plantarum* WCSF1 has been extensively studied, up to the level of genome scale modelling and growth optimization (Teusink *et al*., 2005, 2006). In retrospect, these were exactly the attributes why this particular strain was selected for genome sequencing a dozen years ago (Kleerebezem *et al*., 2003). In this review, we present an overview of the main insights that the molecular research on *L. plantarum* WCSF1 has provided in relation to the interaction with the host and potential health benefit for humans. Moreover, we indicate a variety of avenues that can be followed up for future industrial applications of this strain and summarize suggestions further research that is needed for such applications.

## From genome to function

The initial genome sequence of *L. plantarum* WCFS1 was based on Sanger dideoxy‐sequencing (Kleerebezem *et al*., 2003) and has been revised by next generation sequence analysis on an Illumina platform, providing a genome sequence predicted to code for 3042 proteins (18 pseudogenes) and 83 RNA‐encoding genes (Siezen and van Hylckama Vlieg, 2011).

The genome contains two large regions, one of approximately 150 kb (at position 2.70–2.85 Mb) and one of 190 kb (at position 3.10–3.29 Mb) that are variable in many other *L. plantarum* strains and have been termed life style islands, since they code for a total of 293 genes that are mainly involved in sugar degradation. (Molenaar *et al*., 2005). Furthermore, *L. plantarum* WCFS1 contains three plasmids, including two small ones, pWCFS101 and pWCFS102, that are rolling‐circle replicating plasmids with an unclear function and a size of 1917 and 2365 bp respectively. A third plasmid, pWCFS103 has a size of 36 069 bp, the capacity for conjugative transfer, and encodes genes involved in heavy‐metal resistance (cadmium and arsenate) and NADH oxidase activity (Van Kranenburg *et al*., 2005).

The genome of *L. plantarum* WCFS1 has been well annotated not only by automated methods but also by detailed manual curation. However, there is still a large fraction (approximately 30%) of genes for which no function can be predicted. Moreover, as is the case with all genomes, in some cases the annotated genes are not correctly predicted and a combination of genetic and physiological experiments is needed to demonstrate the functionality of a gene. Due to the high transformation efficiency of *L. plantarum* WCFS1 (De Vos, 2011), a variety of useful inactivation systems (Lambert *et al*., 2007), and controlled expression platforms (such as NICE; Pavan *et al*., 2000), a great number of isogenic mutants have been generated in *L. plantarum* WCFS1 or NCIMB 8826. Many of these are relevant for its growth, cell shape or surface properties and its interactions with the environment – hence these are listed here and some of these are discussed further (see Table 1). Apart from the genetic systems, a useful set of high throughput tools have been applied in recent years, varying from various microarray platforms, RNAseq approaches and advanced proteomics (Molenaar *et al*., 2005; Bron *et al*., 2006; Cohen *et al*., 2006). This allowed detailed phenotypic analysis of mutants, functional studies of overexpressed genes, and the evaluation of genome‐wide expression in response to environmental cues. Many of these approaches have been instrumental in not only confirming the predicted function of genes but also defining new and relevant properties, notably for gastrointestinal (GI) tract survival and interactions with food, other bacteria and the host, as will be discussed below.

**Table 1:** Overview of relevant *Lactobacillus plantarum* WCFS1 mutants, the involved gene and their phenotypes, classified according to their gene function. Some mutants with mutations in homologous genes and similar phenotypes are combined

Gene(s)	Locus	Gene function	Affected phenotype	Reference
Substrate utilization & respiration				
*melA*	lp_3485	α‐Galactosidase	Melibiose utilization	Lambert *et al*. (2007)
*lacS2*	lp_3468	Sugar permease	ND	Lambert *et al*. (2007)
*cydA*	lp_1125	Subunit cytochrome (bd type)	Oxidative respiration	Brooijmans *et al*. (2009)
*narG*	lp_1497	Subunit nitrate reductase	Nitrate respiration	Brooijmans *et al*. (2009)
*rpoN*	lp_0787	Sigma factor 54	Global expression	Stevens *et al*. (2010)
*manR*	lp_0585	Mannose operon regulator	Mannose utilization	Stevens *et al*. (2010)
*manIIC*	lp_0576	Mannose transport	Mannose utilization	Stevens *et al*. (2010)
*ccpA*	lp_2256	Carbon control protein	Glucose repression	Zotta *et al*. (2012)
*lpdB*	lp_0271	Gallate decarboxylase	Tannine utilization	Jiménez *et al*. (2013)
*lpdC*	lp_2945	Gallate decarboxylase	Tannine utilization	Jiménez *et al*. (2013)
Quorum sensing & bacteriocin production				
*lamA*	lp_3580	Response regulator	QS/EPS/biofilm production	Sturme *et al*. (2005)
*lamR*	lp_3087	Response regulator	QS/EPS/biofilm production	Fujii *et al*. (2008)
*plnGHSTUVWX*	lp_0423‐0430	QS pheromone & transport	QS pheromone production	Meijerink *et al*. (2010)
*plnEFI*	lp_0419‐0423	ABC transporter	Plantaricin transport	Meijerink *et al*. (2010)
*plnABCD*	lp_0415‐0418	Plantaricin & QS module	Plantaricin A production	Maldonado‐Barragán *et al*. (2009)
Stress response and intestinal tract survival				
*bsh1*	lp_3538	Choloyl glycin hydrolase	Bile resistance	Lambert *et al*. (2007)
*bsh2‐bsh3‐bsh4*	lp_0067‐lp_3362‐lp_2572	Penicillin acylase	Acylase activity	Lambert *et al*. (2008a)
*ctsR*	lp_1018	Class III stressor	Stress/control ftsH expression	Fiocco *et al*. (2009)
*ftsH*	lp_0547	Chaperone protease	Stress resistance	Fiocco *et al*. (2009)
*hsp 18.55*	lp_3352	Heat shock protein	Membrane fluidity	Capozzi *et al*. (2011)
Cell surface proteins & host interaction				
*lp_0373*	lp_0373	Cell Surface protein	ND	Pretzer *et al*. (2005)
*msa*	lp_1229	Mannose‐specific adhesion	Agglutination	Pretzer *et al*. (2005)
*srtA*	lp_0514	Sortase	Protein anchoring	Pretzer *et al*. (2005)
*lp_2940*	lp_2940	Cell surface protein	Mouse GI tract passage	Bron *et al*. (2007)
*lp_1164*	lp_1164	Cellobiose EII transporter	Mouse GI tract passage	Bron *et al*. (2007)
*lp_3055*	lp_3055	Copper transporter	Mouse GI tract passage	Bron *et al*. (2007)
*napA3*	lp_2827	Na/H antiporter	In vitro GI tract survival	Van Bokhorst‐van de Veen *et al*. (2012a)
*pbp2A*	lp_1413	Penicillin‐binding protein	In vitro GI tract survival	Van Bokhorst‐van de Veen *et al*. (2012a)
*lp_1699*	lp_1699	AraC regulator	In vitro GI tract survival	Van Bokhorst‐van de Veen *et al*. (2012a)
Cell shape or surface properties modulation				
*dltD*	lp_2016	D‐alanine transfer	Charged techoic acids	Grangette *et al*. (2005)
*alr*	lp_0523	Alanine racemase	Cell envelope integrity	Palumbo *et al*. (2004)
*acm2*	lp_2645	*N*‐acetyl glucosaminidase	Autolysin cell separation	Rolain *et al*. (2012)
*lytA*	lp_3421	D,L endopeptidase	Cell shape and integrity	Rolain *et al*. (2012)
*lys2*	lp_3093	*N*‐acetylmuramidase	Cell separation	Rolain *et al*. (2012)
*tagF1‐tagF2*	lp_0268‐ lp_0269	Glycerol phosphate transferase	Wall techoic acid modification	Tomita *et al*. (2013)
*cps1A*	lp_1177	SPS production	Reduced SPS and rhamnose level	Remus *et al*. (2012)
*cps2A*	lp_1197	SPS production	Reduced SPS levels	Remus *et al*. (2012)
*cps3A‐cps4A*	lp_1215‐ lp_2108	SPS production	Reduced SPS levels	Remus *et al*. (2012)
*gtfA*	lp_1299	Glycosyl transferase	Surface protein glycosylation	Lee *et al*. (2014)
*gtfB*	lp_1311	Glycosyl transferase	Surface protein glycosylation	Lee *et al*. (2014)
*oatA*	lp_0856	O‐acetyl transferase	Cell septation	Bernard *et al*. (2011)
*oatB*	lp_0925	O‐acetyl transferase	Cell septation	Bernard *et al*. (2011)

GI, Gastrointestinal; EPS, Extracellular polymeric substance; SPS, Surface Poly Saccharide; QS, Quorum Sensing; ND, not detected.

## Gastrointestinal tract survival

*L. plantarum* NCIMB 8826, the parental strain of *L. plantarum* WCFS1, shows high survival capacity in the human GI tract. After a single oral dose of 1.5 × 10^10^ CFU ml^−1^, the survival of *L. plantarum* NCIMB 8826 was 7%, which was much higher than that of *L. fermentum* KLD and *Lactococcus lactis* MG 1363 that showed an ileal survival of 0.5% and 1%, respectively (Vesa *et al*., 2000). On day 7 of a 1‐week ingestion period, *L. plantarum* NCIMB was found at high concentrations of 10^8^ CFU g^−1^ in faecal samples (Vesa *et al*., 2000). In addition, *L. plantarum* WCFS1 was readily obtained from the ileal effluents of ileostoma patients fed an oral dose (Marco *et al*., 2010). Another study demonstrated that *L. plantarum* WCFS1 in healthy human volunteers survived the in vivo GI tract passage, as a 100‐ to 1000‐fold increased level of *L. plantarum* WCFS1 compared with other *L. plantarum* strains could be recovered from faecal samples until 3–4 days after administration (Van Bokhorst‐van de Veen *et al*., 2012b). The relative survival rate of *L. plantarum* WCFS1 under conditions mimicking the GI‐tract passage was high compared with other *L. plantarum* strains (e.g. a difference in survival of 7 log_10_ CFU ml^−1^ compared with *L. plantarum* CECT4646). However, the human volunteer trials confirmed an earlier study (Vesa *et al*., 2000) and indicated that *L. plantarum* WCFS1 is a passenger in the GI tract, and not an effective intestinal colonizer as found for various other Lactobacilli (Douillard and de Vos, 2014). It should be stressed that only the lumen was analysed, whereas the bacteria could have colonized the intestinal epithelium. Furthermore, during the GI passage the microorganism is able to exert its effect on the physiological and immunological systems of the host. For instance, the well‐studied probiotic strain *L. rhamnosus* GG, which has been subscribed to be a very good mucus adhering *Lactobacillus* strain due to mucus‐binding pili (*spa*CBA‐*srt*C1), is also only able to temporary colonize the gut (up to 1 week; Goldin *et al*., 1992; Alander *et al*., 1999; Segers and Lebeer, 2014). It is assumed that the majority of Lactobacilli are passengers in the GI tract, rarely exceeding 1% of the total number of bacteria (Douillard and de Vos, 2014).

The first hurdle a consumed bacterium encounters when entering the GI tract is the acidic stomach. An in vitro GI tract survival model, in which bacteria were exposed for 1 h to gastric juice containing pepsin and lipase at a pH of approximately 2.5, and subsequently subjected to pH‐neutralizing pancreatic juice containing pancreatin and bile salts, *L. plantarum* WCFS1 proved to be robust, with a 3‐log decrease in viable cells (Van Bokhorst‐van de Veen *et al*., 2012a). The gastric juice exerted the highest impact on *L. plantarum* WCFS1 survival, demonstrated by a million‐fold decrease in living cells, whereas the condition resembling the small intestines hardly affected the survival (Van Bokhorst‐van de Veen *et al*., 2012a). Another oro‐gastric‐intestinal tract model demonstrated that survival of *L. plantarum* WCFS1 is unaffected by the initial oro‐gastric stress, however, the viability decreased significantly when pH was downshifted to approximately 2.0 (Bove *et al*., 2013). There are several mechanisms upregulated in response to low‐pH conditions, for instance increased proton export by F_0_F_1_‐ATPase to retain a proper intracellular pH. Furthermore, the gastric stress was associated with an increased expression of the chaperone genes *dnaK*,* groEL*,* clpB* and *clpE*, small heat shock proteins *hsp*1, *hsp*2 and *hsp*3. In addition, the expression of the adhesion factors mucus‐binding (*mub*) and mannose‐specific adhesion (*msa*), and that of the operon *plnEFI*, an ABC transporter involved in plantaricin production, was increased in response to gastric stress (Table 1; Bove *et al*., 2013). The expression levels of three genes (*pbp2A*,* napA3* and *lp_1669*) were negatively correlated with in vitro GI‐survival and encode a penicillin‐binding protein 2A, an Na^+^/H^+^‐antiporter and an AraC family regulator (see Table 1) that may control the expression of surface polysaccharide production respectively (Van Bokhorst‐van de Veen *et al*., 2012a).

After survival of the stomach passage, an ingested bacterium reaches the duodenum, where it encounters a variety of stressful conditions, including the presence of conjugated bile salts. Not only do these bile salts disperse and absorb fat, these compounds also function as surfactants and disrupt the cells membrane integrity, generate free radicals, and when protonated, can lower the intracellular pH (Bron *et al*., 2004a; Van Bokhorst‐van de Veen *et al*., 2012b). Bile salts are deconjugated by bacterial bile salt hydrolases (Bsh) and reabsorbed in the colon. *L. plantarum* WCFS1 contains four genes originally annotated as bile salt hydrolases (*bsh1*,* bsh2*,* bsh3* and *bsh4*; Table 1). However, only Bsh1 was shown to have Bsh activity and can be considered as the major Bsh while Bsh3, 2 and 4 were only able to hydrolyse penicillin V and penicillin G (Lambert *et al*., 2008a,b). However, under selective conditions, Bsh1, Bsh3 and Bsh4 could be able to hydrolyse bile salts (Table 1). It is suggested that Bsh plays a role in bile detoxification, GI persistence, serve a nutritional role and induce membrane alterations (Begley *et al*., 2006).

An artificial GI tract environment, consisting of 0.1% oxgall, a bovine bile salt, only marginally affected growth of *L. plantarum* WCFS1 but its morphology was severely changed, for instance into a less smooth surface (Bron *et al*., 2004a). *L. plantarum* reacted on this physiological stress by upregulating the expression of proteins involved in bile export (*lp_0085*,* lp_2564* and *lp_3160*) and four oxidoreductases and a redox protein to restore the oxidative and redox imbalance (Bron *et al*., 2004a). In addition, the genes *lp_0237* and *lp_0775* were found to be bile‐inducible. Overall, 62 and 28 open‐reading frames are up‐ or downregulated respectively. Among the upregulated genes are the oxidative stress‐associated glutathione reductase and the *metC‐cysK* operon (Bron *et al*., 2006). By reducing the expression of non‐essential membrane proteins, the cell might compensate for the bile‐induced loss of membrane integrity (Bron *et al*., 2006). Proteomic and transcriptomic analysis revealed that *L. rhamnosus* GG and *L. casei* BL23 also have a reduced expression of proteins involved in cell wall function in response to bile stress, suggesting this is a more common trait in LAB (Douillard and de Vos, 2014). *L. plantarum* WCFS1 seems to have a large array of response mechanisms to bile salts. Since apparently only the morphology is altered and not the viability, WCFS1 is able to efficiently cope with this stressful environment. The hydrolysis of bile salts is associated with lowering of serum cholesterol as well as mucin production (Lambert *et al*., 2008a). Recently, Bsh in the GI tract have been implied in providing specific signals that reduce weight gain in mice after a high fat diet (Joyce *et al*., 2014).

Although the colonic lumen, where the majority of the microbiota resides, is deprived of oxygen, oxidative stress can be a problem as the mucosal surface is more oxygen‐rich. In addition, a high osmolarity predominates in the colon (Kleerebezem *et al*., 2010). *L. plantarum* WCFS1 has multiple strategies to respond to oxidative stress, like manganese, glutathione, ascorbate, pyruvate, flavonoids, carotenoids and the action of peroxidases. *L. plantarum* WCFS1 was found to respond to oxidative stress using thioredoxin (TRX), the only active thiol‐reducing system in this strain. Not all Gram‐positive bacteria can synthesize the antioxidant glutathione, therefore, the TRX system is essential for this organism (Serrano *et al*., 2007). The expression of the *trxA2* and *trxB1* genes was increased following oxidative stress and *trxB2* in combination with *trxA2* were involved in reductive stress and a temperature shift (Serrano *et al*., 2007). The *trxB1* gene codes for a thioredoxin reductase, which regenerates oxidized TXR (Arnér and Holmgren, 2000).

Using a special in vivo expression technology system, a set of 72 genes of *L. plantarum* WCFS1 was identified whose promoters were specifically active during mouse GI tract passing (Bron *et al*., 2004b). Many of these genes were predicted to be involved in cell wall anchoring, exporters and metabolism. By inactivating a selection of these (Table 1), it was observed that the GI tract survival of the mutants *Δlp_1164*,* Δlp_2940* and *Δlp_3055* was decreased compared to the control strains, whereas the mutants Δ*lp_1403*, Δ*lp_3281* and Δ*lp_3659* showed no differences (Bron *et al*., 2007). The gene at locus *lp_1164* is suggested to encode a component of the cellobiose transport and could be involved in the host‐specific signalling, that at *lp_2940* encodes an extracellular protein and that at *lp_3055* is important in the copper homeostasis, as it is predicted to encode a copper‐transporting ATPase. These genes thus play an important role in the GI‐survival in mice and while the GI tract of mouse and human differ in architecture, pH and microbial composition, it is possible that these genes also play a role in the survival in the human system.

Administration of *L. plantarum* WCFS1 to a mouse model demonstrated that strains could be retrieved from faecal samples up to 7 days (Van Bokhorst‐van de Veen *et al*., 2013). The GI tract persistence could be increased to over 32 days when isolated faecal strains were re‐administered to the mice (Van Bokhorst‐van de Veen *et al*., 2013). The persistent strains all demonstrated a single nucleotide polymorphism (SNP) in genes coding for membrane associated proteins. The CFUs of *L. plantarum* in the stomach and small intestine remained high for at least 4 h after administration of 2 × 10^10^ CFU *L. plantarum* WCFS1 in mice but thereafter declined to background levels. This amount remains, however, at 1 × 10^9^ CFU g^−1^ tissue for at least 8 h in the caecum and colon (Marco *et al*., 2007). A single intragastric gavage consisting of 1 × 10^9^ CFU *L. plantarum* WCFS1 in GF mice on a chow or ‘western’ diet, showed colonization over the intestinal epithelium (Marco *et al*., 2009); remarkably, a significantly higher colonization in the colon and caecum was achieved when mice were on a chow diet. The host diets were associated with dramatically different transcription profiles. For instance, the western diet, consisting of mainly simple sugars, is restricting growth, demonstrated by 3 to 5‐times lower expression of genes involved in transcription, translation and nucleotide biosynthesis (Marco *et al*., 2009).

## Interaction with food components

Current dietary recommendations include the consumption of fruits and vegetables. In addition to the vitamins and dietary fibre content of these products, it is a source of the polyphenol tannin. Tannins can form indigestible protein complexes and bind heavy metals. Tannins have also been associated with hepatotoxicity and cancer (Jiménez *et al*., 2013). On the other hand, tannins have antimicrobial properties; thereby potentially alter the gut composition. *L. plantarum* is so far the only tannin‐degrading *Lactobacillus* species found in humans and contains tannase (tannin acyl hydrolase; Reverón *et al*., 2013). *L. plantarum* WCFS1 is able to hydrolyse tannin into glucose and gallic acid, a harmful and anti‐nutritional compound, which is decarboxylated by LpdB and LpdC (*lp_0271* and *lp_2945*) that encode gallate decarboxylase activity (Jiménez *et al*., 2013). Other *L. plantarum* strains that are suggested to possess tannase‐activity are *L. plantarum* CNRZ 1228, CNRZ 184, ATCC 8014 and ATCC 14917 (Osawa *et al*., 2000). Culturing of *L. plantarum* WCFS1 in the presence of tannic acid induces significantly higher expression of persistence and survival genes *copA*,* lp_2940*,* ram2* and *argG* that are highly induced in the GI tract in response to high osmolarity and bile in mice and humans (Reverón *et al*., 2013). *L. plantarum* WCFS1 is thus able to respond to these toxic compounds as well as use these as an energy source, thereby selectively stimulating its growth.

Plant cell walls contain many phenolic compounds which, when released, have shown to have several beneficial effects on the host (e.g. anti‐inflammatory and anti‐oxidants; Esteban‐Torres *et al*., 2013). Feruloyl esterase (FE; *lp_0796*), an enzyme involved in the release of these compounds would be able to release these beneficial compounds. Unfortunately, an efficient transport system for feruloyl esters appeared to be lacking in *L. plantarum* WCFS1 as it was unable to hydrolyse any of the extracellular model substrates (Esteban‐Torres *et al*., 2013); cell extracts could, however, partially hydrolyse methyl ferulate and methyl p‐coumarate, demonstrating that the enzyme Lp_0769 is functional and is likely to be released upon lysis of *L. plantarum* WCFS1. Whether the activity of FE in *L. plantarum* WCFS1 is of significance remains to be determined, as other strains such as *L. fermentum* NCIMB 5221 and *L. fermentum* 11976 have superior FE‐activity and already demonstrate potential health effects (Bhathena *et al*., 2009; Tomaro‐Duchesneau *et al*., 2012).

*L. plantarum* WCFS1 encodes a p‐nitrobenzoate reductase (PnbA; encoded by *lp_0050*). The PnbA enzyme catalyses the reduction in nitroaromatics which are highly abundant food products due to several industrial processes (Guillen *et al*., 2009). These nitroaromatic compounds have been shown to be cytotoxic and mutagenic, therefore bacterial nitroreductases can have beneficial health effects on the host. PnbA is a highly selective reductase as it only reduces 4‐nitrobenzoate and 2,4‐dinitrobenzoate (Guillen *et al*., 2009).

Currently, many commercial products contain prebiotics, which are substances that selectively stimulate growth and/or activity of one or a limited number of bacteria and can thereby be beneficial to the host (Gibson *et al*., 2004). Short chain fructooligosaccharides (scFOS), a well‐studied prebiotic, is converted by *L. plantarum* WCFS1 by a sucrose phosphoenolpyruvate transport system, a β‐fructofuranosidase and a fructokinase (Saulnier *et al*., 2007). In vitro culturing indicated that scFOS are the optimal prebiotic substrates, although growth on scFOS was relatively slow. Detailed analysis showed that preferentially the trisaccharide 1‐ketose is used and its conversion was heterofermentative, since the end‐products are mainly lactate and acetate.

## Interaction with other microorganisms

For successful GI tract survival, adaptation and response to environmental cues, *L. plantarum* needs sensing to react to other, mutualistic and competing, microorganisms. Gene expression depending on cell‐density is referred to as quorum sensing, and can significantly aid in the survival of the bacteria (Kleerebezem *et al*., 1997; Sturme *et al*., 2007). The quorum sensing systems are regulated by signal molecules, autoinducing peptides (AIPs) that are sensed by a two component systems (TCS), that include a histidine protein kinase (HPK) and a response regulator (RR; Sturme *et al*., 2005). The quorum‐sensing systems of *L. plantarum* WCFS1 have been well‐studied, notably the quorum‐sensing systems for the production of bacteriocins. *L. plantarum* WCFS1 possesses the *pln* locus that contains five operons; *plnABCD*, that encodes the AIP termed plantaricin A (*plnA*) which also is a bacteriocin, the HPK PlnB (*plnB*) and the two RRs PlnC and PlnD (Table 1; Sturme *et al*., 2007). *L. plantarum* WCFS1 shares this locus (to some extend) with *L. plantarum* C11, NC8 and J23 (Rojo‐Bezares *et al*., 2008). At a certain bacterial cell density, the plantaricin A concentration reaches a threshold, thereby activating the HPK PlnB and this subsequently phosphorylates the RRs PlnC an PlnD. The RRs regulate the transcription of all the genes involved in bacteriocin synthesis. In this way, there is a density‐dependent expression of plantaricin A. The operons *plnEFI* and *plnJKLR* encode the plantaricins EF and JK with their respective immune proteins (Rojo‐Bezares *et al*., 2008). The bacteriocins are subsequently transported and secreted by an ABC transporter and accessory proteins (PlnGH) encoded by the operon *plnGHSTUVWXY*, the role of which remains to be determined (Sáenz *et al*., 2009).

Bacteriocins play an important role in the competition with other microorganisms. Based on their characteristics, bacteriocins are distinguished into several classes. The antimicrobial peptides of *L. plantarum* WCFS1 can be classified into the non‐lantibiotic family (class II) and include the well‐studied plantaricin A, which is a class IIc bacteriocin, and the plantaricins EF and JK belonging to the class IIb two‐peptide bacteriocins (Table 1; Diep *et al*., 2009). The antimicrobial activity of plantaricin A has a relatively narrow spectrum and is significantly lower activity than that of the plantaricins EF and JK (Diep *et al*., 2009). The latter bacteriocins PlnEF and PlnJK are mostly active against *Lactobacillus* species and closely related Gram‐positive bacteria (e.g. *L. plantarum*,* L. casei*,* L. sakei*,* L. curvatus*,* Pediococcus pentosaceus* and *P. acidilactici*), whereas plantaricin A is effective against *Lactobacillus* species, such as *L. casei*,* L. sakei*,* L. plantarum* and *L. viridescens* (Diep *et al*., 2009). *L. plantarum* WCFS1 demonstrated bacteriocin production with a low minimum inhibitory concentration against *Enterococcus faecalis* CNRZ135, *L. pentosus* CECT4023T, *L. plantarum* CECT748T, *Listeria innocua* BL86/26 and *P. pentosaceus* FBB63. The bacteriocin production however depends on the inoculation size and is dependent on quorum sensing as described above. Hence, at low cell densities, the bacteriocin production is too low to inhibit growth of competing microorganisms (Maldonado‐Barragán *et al*., 2009). Moreover, the bacteriocins have not shown to be active against clinically relevant Gram‐positive pathogens, such as *Staphylococcus aureus* or *Listeria monocytogenes*, and therefore, their medical or biotechnological application remains limited.

Quorum sensing is also essential in the formation of biofilms. Biofilms render the bacteria less sensitive to antimicrobials due to reduced penetration and resistance mechanisms. In addition, bacteria are less susceptible due to a lower growth rate (Van der Veen and Abee, 2011). For instance, cells of *L. plantarum* WCFS1 in a mixed biofilm with *L. monocytogenes* were more resistant to disinfection treatments by benzalkonium chloride and peracetic acid than the single species biofilms (Van der Veen and Abee, 2011). The formation of biofilms with other species might be beneficial to the host as it may involve co‐aggregation with pathogens, thereby decreasing the colonization potential of the pathogens (Goh and Klaenhammer, 2010). Auto‐aggregation entails the aggregation of genetically identical cells, and can enhance the resistance to stress in the intestines (Hevia *et al*., 2013). Aggregation promoting factors (APFs) are extracellular proteins, highly expressed in the stationary phase, that are directly linked to the ability to co‐aggregate (Boris *et al*., 1997). In *L. plantarum* NCIMB 8826, the serine/threonine domain of the APF, D1, binds to (porcine) mucin III and is involved in auto‐aggregation as it has been found that *L. plantarum* loses its auto‐aggregative abilities when gene D1 is knocked‐out (Hevia *et al*., 2013). Moreover, gene D1 overproduction in *L. lactis* leads to aggregation. As *L. plantarum* WCFS1 has been derived from strain NCIMB 8826, it was not a surprise to find the gene D1 in the *L. plantarum* WCFS1 genome as Lp_0304, which has been annotated as an extracellular transglycosylase. However, gene D1 contained several SNPs as compared to the known sequence of Lp_0304 (Kleerebezem *et al*., 2003), either reflecting sequence errors or strain heterogeneity in NCIMB 8826, but it is likely that Lp_0304 also can bind mucus as it has the serine/threonine domain.

The accessory gene regulatory system performs a key role in biofilms formation and the *lamBDCA* operon of *L. plantarum* WCFS1 controls the expression of around 100 genes (Sturme *et al*., 2005). This system encodes a HPK (LamC) and the RR (LamA) that form a TCS, as well the AIP (LamD) and the export and modification protein (LamB). The expression of the *lamBDCA* operon seems to correlate with growth, as expression increased during the exponential phase. The AIP was found to be a novel cyclic thiolactone AIP that seems to control adherence, most likely via its effect on the expression of EPS operons (Sturme *et al*., 2005). The *lamBDCA* operon was found to be engaged in cross‐talk with another TCS encoded by the *lamKR* operon, as a *lamA/lamR* mutant demonstrated a highly reduced adherence to glass compared to a single mutant or wild‐type (Fujii *et al*., 2008). TCSs monitor and respond to environmental cues such as stress (Sturme *et al*., 2007). Quorum sensing is thus essential in the formation of biofilms, which renders *L. plantarum* WCFS1 less susceptible to external stressors due to restricted penetration.

Peptidoglycan hydrolases (PGHs) are major actors in cell division, cell wall turn over, autolysis and biofilm formation. By cleavage of the bacteria peptidoglycan, they may even play a role in host interaction by the release of muramyl‐peptides and PG fragments (Rolain *et al*., 2012). The genome of *L. plantarum* WCFS1 encodes for at least 12 PGHs, with *N*‐acetylglucosaminidase (Acm2) and γ‐d‐Glu‐mDAP muropeptidase (LytA) as the most pivotal proteins for physiology and morphogenesis (Rolain *et al*., 2012; Table 1). It has recently been observed that Acm2 is post‐translationally modified by glycosylation (Fredriksen *et al*., 2012) and this may further enhance the interaction with bacteria, contributing to biofilm formation.

## Interactions with the host – epithelial barrier

**Figure 1:**
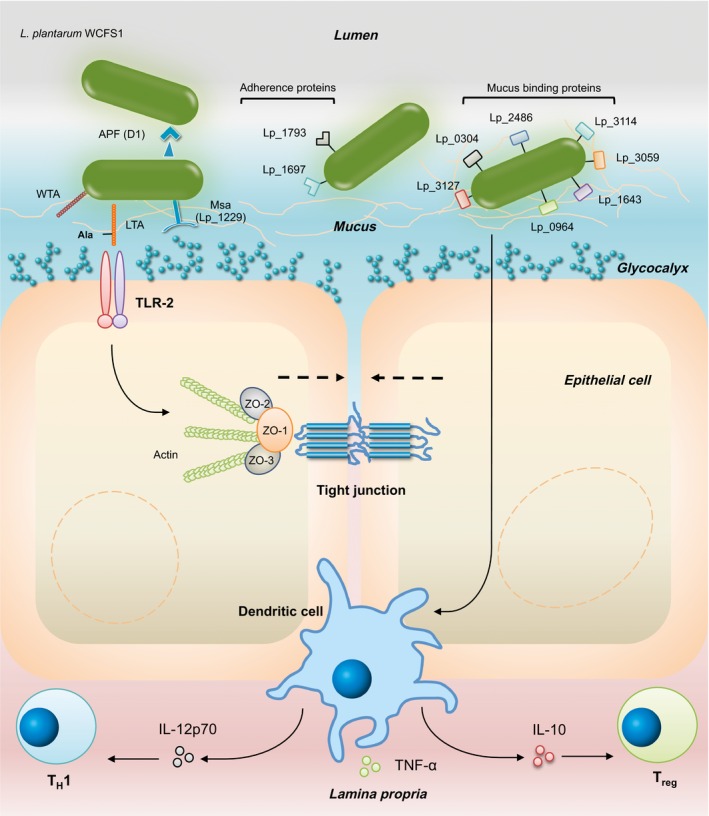
Putative proteins involved in the host–microbe interaction of *Lactobacillus plantarum* WCFS1. APF(D1), Aggregation promoting factor D1; WTA, Wall teichoic acid; LTA, Lipoteichoic acid; TLR, Toll‐like receptor; Msa, Mannose‐specific adhesion; ZO‐1, Zonulin‐1; ZO‐2, Zonulin‐2; ZO‐3, Zonulin‐3; IL, Interleukin; TNF, Tumour necrosis factor; T_H_1, T‐helper cell 1; T_reg_, Regulatory T cell.

Extracellular proteins, that together constitute the secretome, are involved in variable processes such as host‐adherence, recognition, degradation and uptake of luminal nutrients and transduction of signals (Buck *et al*., 2005). The genome of *L. plantarum* WCFS1 encodes 223 predicted extracellular proteins of which 81 are membrane anchored, 37 attached to the cell wall, 48 covalently bound through a lipobox and 57 have been predicted to be secreted (Boekhorst *et al*., 2006). Analysis of the secretome identified 12 adhesion factors; three contained a domain to adhere to collagen, one to chitin, one to fibronectin and seven to mucus. *L. plantarum* WCFS1 also contains a MUB domain, a domain that is unique for LAB and present in the MUB products of four genes (*lp_1229*,* lp_3114*,* lp_3059* and *lp_1643*; Boekhorst *et al*., 2006; Fig. 1). These include *lp_1229*, which encodes the Msa (Table 1). Deletion of the *msa* gene resulted in loss of the ability of *L. plantarum* WCFS1 to agglutinate with yeast (Pretzer *et al*., 2005). When comparing to other *Lactobacillus* strains, 14 mucus‐binding proteins were identified in *L. gasseri* ATCC 33323, and 18 proteins with potential adhesive properties in *L. acidophilus* L‐92 (Douillard and de Vos, 2014); demonstrating the large repertoire of adhesive proteins within LAB.

*L. plantarum* encodes 32 proteins with a LPxTG‐motif, which are proteins that are covalently bound to the cell wall as they are recognized and cleaved by sortase A, the product of the *srtA* gene. In Gram‐positive pathogens, these LPXTG motif‐containing proteins are often virulence factors, and associated with functions as adhesion and receptors. In some cases, these proteins are post‐translationally glycosylated and are suggested to play a major role in cell‐to‐cell interaction (Fredriksen *et al*., 2013). O‐linked glycosylated extracellular proteins are of interest due to their matrix interaction. For instance, the *L. plantarum* WCFS1 major autolysin Acm2 and the MUB protein lp_1643 (see above), are O‐linked glycosylated (Fredriksen *et al*., 2013). Our current understanding of glycosylation in LAB is limited, but these glycoproteins likely play a role in the bacteria‐host interaction (Fredriksen *et al*., 2012; Tytgat and Lebeer, 2014).

An important site for bacteria‐host interaction is the GI epithelial cells that form a barrier between the body and the inside of the lumen of the gut. An increased or altered GI permeability of this barrier is associated with a variety of illnesses. It has been suggested that inflammatory bowel diseases (IBD) and irritable bowel syndrome (IBS) and IBS are characterized by infiltration of antigens due to mucosal barrier dysfunction and subsequent ongoing inflammation of the intestines (Barbara, 2006; Bruewer *et al*., 2006). The epithelial integrity is mainly controlled by tight junctions (TJs), which are multifunctional complexes of integral membrane proteins located at the apical parts of the epithelial cell (Schneeberger and Lynch, 2004). These structures interconnect the cells and include occludins, claudins and junction adhesion molecules. *L. plantarum* WCFS1 has the potential to enhance the intestinal integrity in cell lines through activation of toll‐like receptor (TLR)‐2, which is expressed on intestinal epithelial cells (Karczewski *et al*., 2010). In vitro activation of TLR‐2 transiently enhanced the epithelial resistance through zonula occludens 1 (ZO‐1) translocation (Cario *et al*., 2004). An experiment using a Caco‐2 human epithelial model demonstrated a significant translocation of ZO‐1 to the TJ‐region due to *L. plantarum* WCFS1 (Fig. 1). This was also observed in the duodenum of healthy individuals after short‐term (6‐h period) administration of *L. plantarum* WCFS1, suggesting that this also occurs in humans (Karczewski *et al*., 2010). In addition, the drop in trans epithelial electrical resistance (TER) induced by phorbol 12,13‐dibutyrate, which dislocates occludin and ZO‐1, was decreased in combination with *L. plantarum* WCFS1 (Karczewski *et al*., 2010). The enhanced barrier function is likely due to an altered TJ composition rather than an increase in TJ proteins, as transcription levels were not significantly altered by *L. plantarum* WCFS1 (Troost *et al*., 2008). Other *L. plantarum* strains were also able to prevent a reduction in TER in Caco‐2 cells when co‐cultured with pathogenic *Escherichia coli* strains (Ulluwishewa *et al*., 2011), indicating that *L. plantarum* can play a beneficial role in maintaining epithelial integrity.

Lipoteichoic acid, major constituents of the cell wall of Gram‐positive bacteria (and suggested as equivalent of the Gram‐negative lipopolysaccharides (LPS)), are important molecules for interaction with TLR‐2. The inflammatory properties of LTA greatly depend on the decoration of this protein by ᴅ‐Ala. A *L. plantarum* NCIMB 8826 Dlt^−^ mutant that results in LTA with significantly less incorporated ᴅ‐Ala units induces significantly less pro inflammatory cytokines when incubated with peripheral blood mononuclear cells (PBMCs) (Grangette *et al*., 2005). In addition, deletion of *lp_2991*, a repressor of the LTA glycosylation enzyme Gtca3, led to significant higher interleukin (IL)‐10, IL‐12p70 and tumour necrosis factor (TNF)‐α levels (Meijerink *et al*., 2010). In agreement, a ᴅ‐Ala mutant in *L. rhamnosus* GG or complete removal of LTA in *L. acidophilus* NCFM led to strongly reduced pro‐inflammatory responses (Segers and Lebeer, 2014). Effects on the epithelial barrier have not yet been investigated for these mutants.

## Interaction with the host – immune systems

In vitro as well as in vivo research has shown immune modulatory capacities for *L. plantarum* WCFS1. Co‐culture of PBMCs with *L. plantarum* NCIMB 8826 showed significant increases in different markers of activated T cells (Dong *et al*., 2012). *L. plantarum* WCFS1 induces expression of different pro‐inflammatory cytokines as well as the anti‐inflammatory cytokine IL‐10 by PBMCs (Larché *et al*., 2003; Van Hemert *et al*., 2010; Dong *et al*., 2012), although the concentration of induced IL‐10 and IL‐12 were relatively low and moderate compared to other *L. plantarum* strains (Van Hemert *et al*., 2010). Co‐culture of immature monocyte derived dendritic cells (DCs) with *L. plantarum* WCFS1 activated the DCs and induced expression of the cytokines IL‐10, TNF‐α and the T_H_1 inducing cytokine IL‐12p70 (Larché *et al*., 2003; Smelt *et al*., 2012; Remus *et al*., 2013). A more IL‐10/IL‐12 cytokine profile would be beneficial in an allergic and autoimmune disorder; however, one should wonder whether these subtle changes will lead to significant effect in vivo. Genes of *L. plantarum* WCFS1 involved in immunomodulation include an N‐acetyl‐glucosamine/galactosamine phosphotransferase system, the LamBDCA quorum sensing system, components of the plantaricin (bacteriocin) biosynthesis and transport pathway, and transcription regulator *lp_2991* (Meijerink *et al*., 2010; Van Hemert *et al*., 2010). However, the function of these genes is quite different and hence it is likely that different mechanisms underlie the observed phenotypes. Moreover, no human data are available as the mutants are generated by genetic modification (GMO), precluding human trials. Non‐GMO approaches as recently described for *Lactobacillus rhamnosus* GG and coupled to next generation sequencing may be used to overcome this and provide avenues for human trials to address cause–effect relations (Rasinkangas *et al*., 2014).

In healthy wild‐type mice, *L. plantarum* WCFS1 leads to an increase in the number of regulatory DCs and regulatory T cells in the spleen (Smelt *et al*., 2012). In the small intestine a decrease in the Th1/Th2 ratio was seen, whereas in the large intestine a more regulatory phenotype was induced (Smelt *et al*., 2013a; Fig. 1). Some of these effects were dependent on the D‐alanylation of teichoic acids, as the *L. plantarum* WCFS1 induced immune changes were not observed when the D‐alanylation negative mutant dltX‐D was used (Smelt *et al*., 2013b). Also a human cross‐over study with healthy volunteers indicated establishment of immune tolerance (Van Baarlen *et al*., 2009). The volunteers consumed *L. plantarum* WCFS1 every half an hour for 6 h and thereafter gene expression responses in the duodenal cells were investigated. Among the regulated genes were numerous genes involved in immune regulation. Although this extensive administration does not reflect a ‘real life’ setting, it provides insightful biological context (Van Baarlen *et al*., 2009). Induction of a regulatory phenotype can dampen inflammatory conditions, for instance, such as observed in UC. Indeed, in a murine TNBS‐induced colitis model, administration of NCIMB 8826 led to a dose‐dependent protection level in weak to moderate colitis (Foligné *et al*., 2006).

The effect of *L. plantarum* WCFS1 on the healthy intestinal mucosa transcriptional response was assessed after a short 1 and 6‐h exposure in human volunteers. In a randomized, placebo controlled, cross‐over study 15 healthy individuals were exposed to 1 × 10^11^ CFU *L. plantarum* WCFS1 after which duodenal samples were taken (Troost *et al*., 2008). A 1‐h exposure demonstrated an upregulation of genes involved in the complement pathway (Troost *et al*., 2008). At the same time, genes involved with lipid and fatty acid metabolism, and the major transcriptional regulators were downregulated. Initial contact between *L. plantarum* WCFS1 seems to down‐regulate the proliferation and there is a primary immune response induced to the microbial presence (Troost *et al*., 2008). In agreement, in a comparable set‐up using *L. rhamnosus* GG the mucosal response was characterized by induction of T_H_1 development (Van Baarlen *et al*., 2011). A prolonged exposure of 6 h is associated with upregulation of lipid/fatty acid metabolism and oxidative stress. In addition, genes involved in the antigen presentation are upregulated (Troost *et al*., 2008). These data suggest that the mucosa is initially alarmed, but after 6 h return to their non‐inflammatory proliferative state (Troost *et al*., 2008). No inflammatory signals were expressed at both time‐points.

Studies with *L. plantarum* WCSF1 have shown variable results in the area of allergic diseases, possibly due to the nature of the different antigens. *L. plantarum* NCIMB 8826 dampened the response of DCs derived from house dust mite allergic individuals stimulated with the dust mite allergen Der‐p1 (Pochard *et al*., 2005). However, in a mice study using a well‐established pathogen‐free mouse peanut sensitization model, administration of *L. plantarum* WCFS1 increased the peanut‐extract specific IgG1, IgG2, IgE and mouse mast cell protease‐1 levels in serum significantly (Meijerink *et al*., 2012).

## Conclusions and future directions

During the last 10 years, a large number of mutants of *L. plantarum* WCFS1 have been made by scientists to investigate effects of single or multiple genes (Table 1). Most of these mutants have been studied in only one or a few screening assays and it would be interesting to investigate these mutants in other assays with a focus on host‐microbe interactions. As indicated above, non‐GMO mutants can now be generated and characterized much faster than before using high throughput sequencing (Rasinkangas *et al*., 2014; Derkx *et al*., 2014). Using these and other non‐GMO mutants in human studies, further insight into mechanisms of host‐microbe interaction could be obtained.

*L. plantarum* WCFS1 is unique because of the large amount of molecular studies performed with this strain. This has advanced our knowledge significantly and indicated several properties that could be further exploited, such as effects on epithelial barrier function, stimulation of Th1 cells, and the potential to remove cholesterol. An important safety aspect of probiotic strains is the absence of transferable antibiotic resistant carriers (Bories *et al*., 2008). No transferable antibiotic resistance genes have been identified in the genome of *L. plantarum* WCFS1 (Kleerebezem *et al*., 2003). Therefore, it is unlikely that the strain can transfer antibiotic resistance genes. The parental strain is of human origin, and *L. plantarum* has the Qualified Presumption of Safety (of the European Food Safety Authority, EFSA) and Generally Recognized as Safe (of the U.S. Food and Drug Administration, FDA) status. Current probiotics have an excellent safety profile (even in highly immunocompromised individuals), it is unlikely that safety issues around *L. plantarum* WCFS1 will arise (Van den Nieuwboer *et al*., 2014a,b, 2015). Nevertheless, investigators should remain aware of potential risks when administering high dosages to immune compromised individuals. Whether *L. plantarum* WCFS1 can be developed into a successful probiotic remains to be determined and clinical trials showing a health benefit will be necessary.
